# Chasing a rarity: a retrospective single-center evaluation of prognostic factors in primary gliosarcoma

**DOI:** 10.1007/s00066-021-01884-0

**Published:** 2021-12-22

**Authors:** Cas S. Dejonckheere, Alexander M. C. Böhner, David Koch, Leonard C. Schmeel, Ulrich Herrlinger, Hartmut Vatter, Matthias Schneider, Patrick Schuss, Frank A. Giordano, Mümtaz A. Köksal

**Affiliations:** 1 Department of Radiation Oncology, University Hospital Bonn, Venusberg-Campus 1, 53127 Bonn, Germany; 2 Institutes for Molecular Medicine and Experimental Immunology, University Hospital Bonn, Bonn, Germany; 3 Division of Clinical Neuro-Oncology, Department of Neurology, University Hospital Bonn, Bonn, Germany; 4 Department of Neurosurgery, University Hospital Bonn, Bonn, Germany

**Keywords:** Gliosarcoma, Glioblastoma multiforme, Brain tumor, IDH-wildtype, MGMT promoter, Radiotherapy, Temozolomide

## Abstract

**Background and purpose:**

Primary gliosarcoma (GS) is a rare variant of *IDH*-wildtype glioblastoma multiforme. We performed a single-center analysis to identify prognostic factors.

**Patients and methods:**

We analyzed the records of 26 patients newly diagnosed with primary WHO grade IV GS. Factors of interest were clinical and treatment data, as well as molecular markers, time to recurrence, and time to death.

**Results:**

Median follow-up was 9 months (range 5–21 months). Gross total resection did not lead to improved survival, most likely due to the relatively small sample size. Low symptom burden at the time of diagnosis was associated with longer PFS (*P* = 0.023) and OS (*P* = 0.018). Median OS in the entire cohort was 12 months. Neither *MGMT* promoter hypermethylation nor adjuvant temozolomide therapy influenced survival, consistent with some previous reports.

**Conclusion:**

In this retrospective study, patients exhibiting low symptom burden at diagnosis showed improved survival. None of the other factors analyzed were associated with an altered outcome.

## Highlights

- Data on prognosis and prognostic factors in gliosarcoma are scarce.
- The median OS in this single-center study was 12 months.
- Patients exhibiting low symptom burden at diagnosis showed improved survival.
- Neither *MGMT* promoter hypermethylation nor adjuvant treatment with temozolomide had an impact on survival.

## Introduction

Gliosarcoma (GS) is a rare variant of glioblastoma multiforme (GBM), accounting for about 2% of cases [1–3]. It can be divided into primary (de novo) and secondary (after prior GBM treated with radiation) GS [4, 5]. It is typically diagnosed in the fifth or sixth decade and is about twice as common in men [2, 5, 6]. GS is usually located in the supratentorial region, with a predilection for the temporal lobe [5, 7, 8]. Presenting symptoms include signs of raised intracranial pressure (e.g., headaches, nausea, and vomiting), visual disturbances, or seizures [5, 9]. Histopathologically, it is characterized by a biphasic growth pattern, including both a glial and an atypical sarcomatous component, descending from a monoclonal origin [10]. In the latest WHO classification, it is regarded as a subtype of the isocitrate dehydrogenase (*IDH*) wildtype GBM, although *IDH*-mutated GS has been described [1, 11].

Because of its only sporadic occurrence, GS is treated in a similar manner to classical GBM, with a multimodal therapeutic approach including maximal safe surgical resection, external beam radiotherapy, and temozolomide-based chemotherapy [12–14]. A historic cohort showed a median overall survival (OS) of only 4 months if left untreated [15]. With treatment, GS continues to have a poor prognosis, with a median OS around 15 months comparable to that of GBM, thus making it one of the tumors of the central nervous system with the lowest relative survival rate [1, 7, 16–18].

In the current study, we present a cohort of 26 GS patients treated at a single center, reviewing their demographic, clinical, and treatment characteristics (16 distinct factors in total). Conclusively, we aim to identify possible independent factors that might be related to an improved outcome.

## Patients and methods

### Patients

**Fig. 1:**
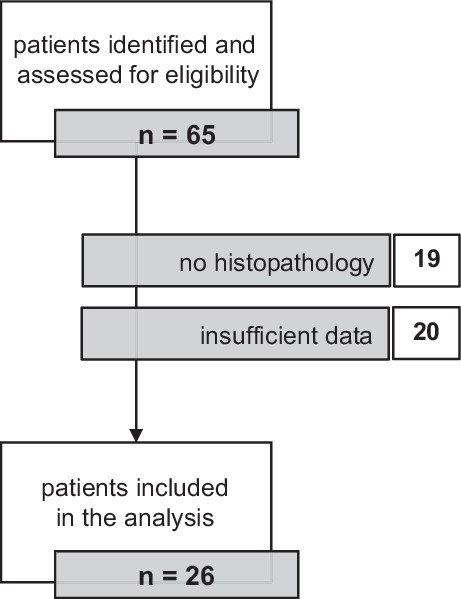
Flowchart of patient selection

An overview of patient selection is presented in Fig. 1. Using structured query language (SQL), the clinical database was searched for cases of GS between January 1995 and May 2021. All matching patient records were analyzed and cases with available information and histopathologically confirmed (by reference neuropathology) GS were included. The following data were extracted: age at diagnosis, gender, tumor location, *MGMT* promotor methylation status and *IDH* mutational status (where available), presenting symptoms and duration, size, surgical treatment (including extent of resection), first-line radiotherapy, first-line chemotherapy, complications, time to recurrence, and time to death. This study was approved by the local ethics committee.

### Treatment and follow-up

Patients were treated per institutional standards, following interdisciplinary case discussion. In the majority of cases, the postoperative Stupp regimen was indicated, consisting of fractionated focal irradiation in daily fractions of 2 Gy given 5 days per week for 6 weeks for a total of 60 Gy, with continuous daily temozolomide during radiation treatment, followed by up to six cycles of adjuvant temozolomide [12]. Older or unfit patients received a reduced radiation dose, with 2.67 Gy 5 days per week for 3 weeks, for a total of 40.05 Gy.

Therapy was monitored weekly during treatment. Upon completion, patients underwent regular assessment of their neurologic status. The first follow-up imaging was scheduled at 6 weeks after completion of the radiation course, then every 3 months, or sooner if indicated.

### Statistical analysis

To identify dependencies of factors on characteristics specific to GS patient data (such as tumor size), linear regressions between pairs of parameters were calculated. Goodness of fit of the regression was provided as R^2^. Statistical dependency of a pair of parameters was assumed if the slope deviance from zero of the regression yielded a *P* < 0.05. For all other relevant factors, a multivariate survival analysis (Cox regression) was performed. For the comparison of outcomes (i.e., survival) by patient group presorted, e.g., by their *MGMT* promotor methylation status or *IDH* mutational status, the Kaplan–Meier survival estimate was used together with the logrank test to generate *P*-values. Patients were censored at the time of death or last follow-up. The statistical analysis was carried out using GraphPad Prism (GraphPad Software v.9.1.0, San Diego, CA 92108, USA).

### Literature search and external data obtainment

A literature search was conducted to identify other original cohorts. The following inclusion criteria were applied: published after 2000, at least 20 patients included, and median OS reported. Excluded were cohorts with exclusively secondary GS, review articles with pooled analyses (to eliminate double inclusions), and registry studies (with high patient counts, for demonstrative purposes).

## Results

### Follow-up and patient characteristics

A total of 26 GS patients were identified, all primary, with a median age of 61 years (range 38–84 years; Table 1). The median follow-up for the entire cohort was 9 months (range 5–21 months). Seventeen patients (65.4%) were male (male to female ratio 1.9:1). All GS manifestations were located in the supratentorial region, with a preference for the temporal lobe (10 patients, 38.5%). The median maximum diameter at diagnosis was 4.1 cm (range 1.0–8.0 cm). All patients underwent surgery followed by radiation treatment. Gross total resection (GTR) was achieved in 18 patients (75.0%). Nineteen patients (73.1%) received temozolomide. Molecular genetics revealed *MGMT* promotor hypermethylation in 7 cases (36.8%). All tumors were *IDH1* wildtype.

**Table 1:** Summary of patient and tumor characteristics (*n* = 26)

Characteristic	*n (%)*
*Median age (range) in years*	*61 (38–84)*
*Gender*	
Male	17 (65.4)
Female	9 (34.6)
*Location*	
Right	16 (61.5)
Left	10 (38.5)
Temporal	10 (38.5)
Frontal	4 (15.4)
Occipital	4 (15.4)
Parietal	3 (11.5)
Multiple lobes	5 (19.2)
*MGMT promotor status*	
Unmethylated	12 (63.2)
Hypermethylated	7 (36.8)
Unknown	7 (26.9)
*IDH status*	
Wildtype	16 (100.0)
Mutated	0 (0.0)
Unknown	10 (38.5)
*KPS at diagnosis*	
100	6 (23.1)
90	8 (30.8)
80	6 (23.1)
≤70	6 (23.1)
*Duration of symptoms*	
Acute event	5 (19.2)
Days	6 (23.1)
Weeks	10 (38.5)
Months	5 (19.2)
*Presenting symptoms*	
Single	6 (23.1)
Multiple	20 (76.9)
Headache	11 (42.3)
Visual disturbances	8 (30.8)
Seizure	7 (26.9)
Motor dysfunction	6 (23.1)
Vertigo	5 (19.2)
Cognitive deficit	5 (19.2)
Speech dysfunction	4 (15.4)
Sensory dysfunction	3 (11.5)
Ataxia	3 (11.5)
Mood disorder	3 (11.5)
Isolated cranial nerve dysfunction	2 (7.7)
Coma	1 (3.8)
Other^a^	3 (11.5)
*Median maximum diameter at diagnosis (range) in cm*^*b*^	*4.1 (1.0–8.0)*
*Surgery*	*26 (100.0)*
Gross total resection	18 (75.0)
Near total resection	2 (8.3)
Subtotal resection	4 (16.7)
Unknown	2 (7.7)
*Radiotherapy (first line)*	*26 (100.0)*
30 × 2 Gy	18 (69.2)
*Chemotherapy (first line)*	*20 (76.9)*
Temozolomide	19 (73.1)
CCNU^c^	4 (15.4)
*Median progression free survival (range) in months*	*7 (0–20)*
*Median overall survival (range) in months*	*12 (3–21)*

*MGMT* O-6-methylguanine-DNA methyltransferase, *IDH* isocitrate dehydrogenase, *KPS* Karnofsky performance status, *CCNU* lomustine

^a^Other symptoms include hiccup, emesis, and urinary incontinence

^b^In 1 patient, tumor diameter was unknown

^c^CCNU was always given in combination with temozolomide

### Symptom load

Headache was the most common presenting symptom, occurring in 11 patients (42.3%), followed by visual disturbances (8 patients, 30.8%) and seizures (7 patients, 26.9%). One patient was diagnosed during pregnancy. Extracranial manifestation was not seen, although 1 patient presented with meningeal spread upon recurrence.

### Outcome

At the time of analysis, 6 patients (23.1%) were still alive, and 3 of them (11.5%) had not reached progression. The median progression-free survival (PFS) in this cohort was 7 months (range 0–20 months), with a median OS of 12 months (range from 3–21 months). One patient died during radiation treatment, from an unrelated cause (endocarditis).

**Fig. 2:**
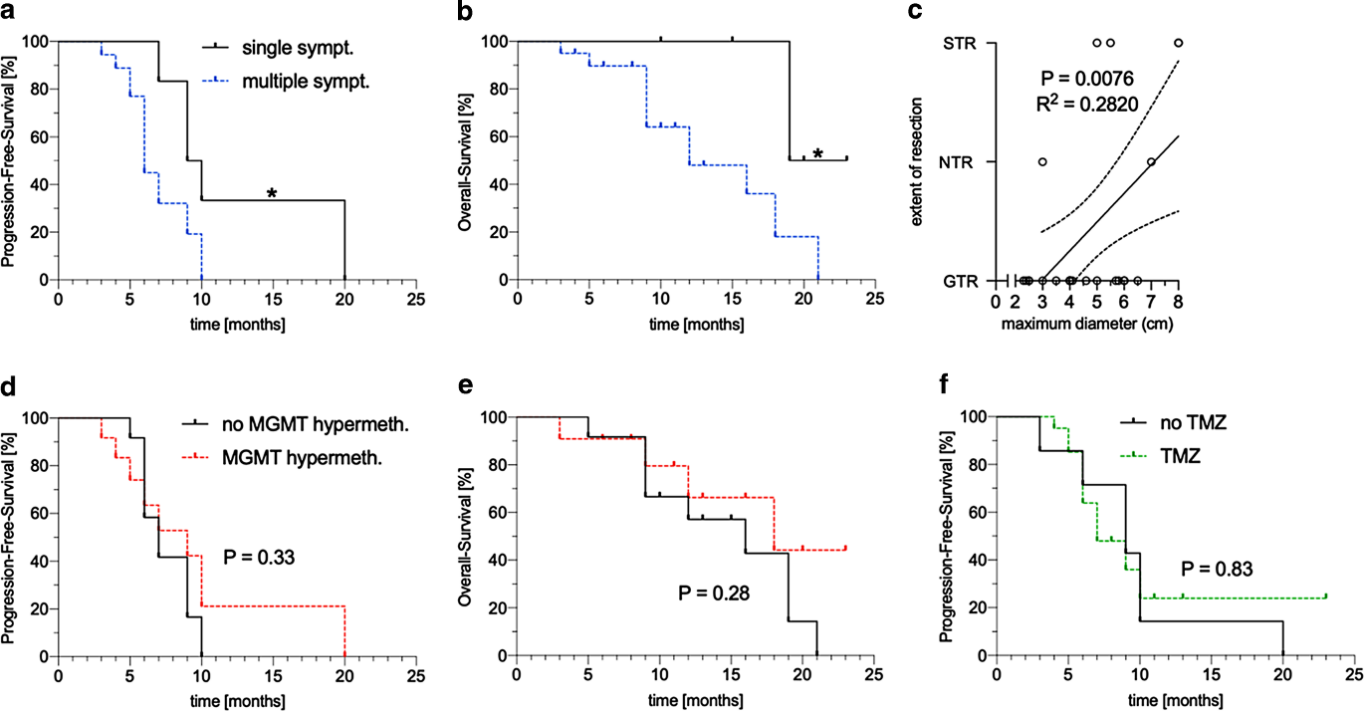
Statistical analysis of the gliosarcoma patient cohort. **a** Dependency of PFS on symptom load (*P* = 0.023). **b** Dependency of OS on symptom load (*P* = 0.018). **c** Correlation between extent of resection and maximum tumor diameter (*P* = 0.0076). **d** Dependency of PFS on *MGMT* promoter methylation status (*P* = 0.33). **e** Dependency of OS on *MGMT* promoter methylation status (*P* = 0.28). **f** Dependency of PFS on TMZ therapy (*P* = 0.83). Significance level *asterisk* for *P* < 0.05. *PFS* progression free survival, *OS* overall survival, *GTR* gross total resection, *NTR* near total resection, *STR* subtotal resection, *MGMT* O-6-methylguanine-DNA methyltransferase, *TMZ* temozolomide

In the statistical analysis (Fig. 2), patients presenting with a single symptom upon diagnosis had better PFS (*P* = 0.023; Fig. 2a) and OS (*P* = 0.018; Fig. 2b). Patients with a smaller tumor were, unsurprisingly, more likely to achieve gross total resection (GTR; *P* = 0.0076; Fig. 2c).

Other variables including age at diagnosis, gender, *MGMT* promotor methylation status (Fig. 2d,e), preoperative performance status, tumor size, extent of resection, and temozolomide-based chemotherapy (Fig. 2f) were not associated with survival.

### Review of literature

**Fig. 3:**
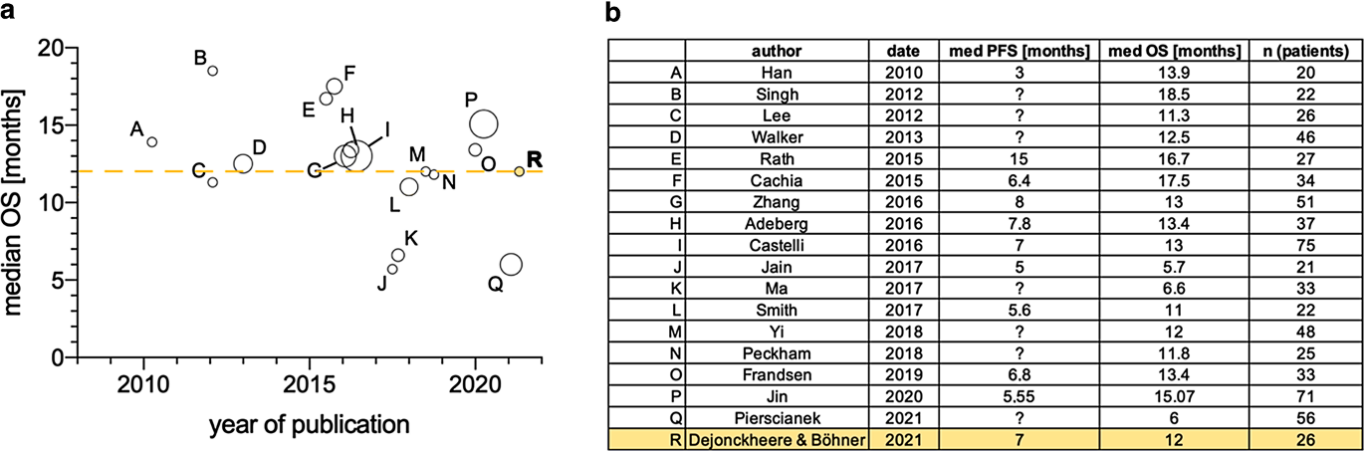
**a** Comparison of median OS in gliosarcoma cohorts published after 2000 with at least 20 patients [4–6, 8, 13, 17–30]. *Bubble size* indicates patient count. **b** There is wide variation in the observed median OS (range 5.7–18.5 months). The present study is highlighted in *yellow* (12 months). The weighted median OS across all studies was 12.9 months. *OS* overall survival, *PFS* progression-free survival

In the literature search, 17 similar original cohorts were identified, accounting for a total of 647 included GS patients [4–6, 8, 13, 17–31]. A comparison of the median OS is displayed in Fig. 3. There seems to be wide variation in the observed median OS between published GS cohorts (range 5.7–18.5 months).

## Discussion

In this retrospective analysis, we present a cohort of 26 GS cases treated at a single center. The demographic and clinical characteristics of our cohort are comparable to previously conducted research [2, 5–7, 9].

Patients presenting with a single symptom upon diagnosis showed improved survival. In 3 of these 6 cases, the symptom was a new-onset seizure in an otherwise healthy individual, warranting prompt investigation, and thus quickly leading to diagnosis and treatment of an intracranial tumor in an early stage. These patients also generally showed a higher Karnofsky performance status.

The extent of tumor resection is a well-established independent prognostic factor for improved OS in GBM [19]. It has also been described in GS [2, 18, 20, 21, 32]. In the current study, GTR did not lead to improved survival. We reason that this might be attributed to the small sample size, since all studies reporting a significant influence on survival had larger patient numbers.

Whether *MGMT* promotor methylation status influences survival in GS remains a matter of ongoing debate. Hypermethylation leads to epigenetic silencing of the *MGMT* gene. Its product, a DNA repair enzyme, restores alkylating agent-induced damage [22]. Hypermethylation of the *MGMT* promotor thus reflects the efficacy of alkylating agents such as temozolomide, and has been thoroughly associated with improved outcome in GBM patients [19]. Reports on the prevalence of *MGMT* promotor hypermethylation and its association with survival in GS are conflicting, however. Some authors reported less frequent hypermethylation in comparison to GBM and used this as an explanation for their observed worse prognosis in GS [7, 23, 32]. In contrast, others suggested more hypermethylation in GS [21, 22]. In the current study, *MGMT* promotor methylation status did not influence survival, consistent with some previous reports [9, 24]. The largest GS registry study to date (with 1102 included patients) found no apparent difference in *MGMT* promotor hypermethylation between GS and GBM, nor did it influence survival in GS patients [18].

Adjuvant temozolomide therapy has become the standard of care in GBM management, leading to a statistically significant and clinically relevant survival benefit [12]. This has also been reported to some degree in GS, albeit mostly in studies with small patient numbers [6, 20, 33, 34]. The largest registry study so far, however, confirmed the superiority of trimodality therapy also in GS [18]. In the current study, there was no statistically significant difference in survival among patients receiving adjuvant temozolomide therapy.

A multitude of other favorable prognostic factors has been reported in the literature, including younger age at diagnosis [2, 18, 23, 25–27, 35], female sex [18], temporal tumor location [7], preoperative performance status [23, 25], and tumor size [23, 26]. These are, however, often not replicated because of small patient numbers, and were also not observed in the present cohort.

The median OS in this cohort was 12 months. There is wide variation in reported median OS in the available literature (Fig. 3; range 5.7–18.5 months). The largest conducted registry study on GS reported a median OS of 10.7 months, comparable to the current cohort [18].

Our study harbors several limitations, most importantly its retrospective nature and relatively small sample size. Furthermore, we did not compare our data to a control group of GBM patients. In earlier cases, information on survival was often missing, which could have been a potential source of bias. However, because of the rarity of GS, we reason that an accurate depiction of GS patients treated at our center is of value and adds to the existing data on GS management.

With a 5-year OS rate of only 5.6%, GS remains a rare tumor entity with a dismal prognosis [17]. Although overlap with GBM exists, GS appears to be a separate tumor entity, with variation in reported prognostic factors. Prospective studies with data on molecular pathology are needed to unequivocally establish such patient and tumor characteristics, aiding patient management and improving care for all GS patients. National and international collaborations could serve useful herein, to facilitate patient recruitment. Until then, a multimodality approach, with surgery aiming for complete resection followed by radiation and chemotherapy, seems to be the most favorable option.
